# Do innate killing mechanisms activated by inflammasomes have a role in treating melanoma?

**DOI:** 10.1111/pcmr.12870

**Published:** 2020-02-20

**Authors:** Abdullah Al Emran, Hsin‐Yi Tseng, Mikaela C. Coleman, Jessamy Tiffen, Stuart Cook, Helen M. McGuire, Stuart Gallagher, Carl Feng, Peter Hersey

**Affiliations:** ^1^ Melanoma Immunology and Oncology Group The Centenary Institute Royal Prince Alfred Hospital University of Sydney Camperdown New South Wales Australia; ^2^ Melanoma Institute Australia The University of Sydney Sydney New South Wales Australia; ^3^ Immunology and Host Defence Group Department of Infectious Diseases and Immunology Sydney Medical School The University of Sydney Sydney New South Wales Australia; ^4^ Tuberculosis Research Program Centenary Institute Camperdown New South Wales Australia; ^5^ Ramaciotti Facility for Human Systems Biology The University of Sydney Sydney New South Wales Australia; ^6^ Discipline of Pathology Sydney Medical School The University of Sydney Sydney New South Wales Australia; ^7^ Charles Perkins Centre The University of Sydney Sydney New South Wales Australia

**Keywords:** adaptive resistance, IL‐1β, inflammasomes, innate killing, melanoma, pyroptosis, TCGA

## Abstract

Melanoma, as for many other cancers, undergoes a selection process during progression that limits many innate and adaptive tumor control mechanisms. Immunotherapy with immune checkpoint blockade overcomes one of the escape mechanisms but if the tumor is not eliminated other escape mechanisms evolve that require new approaches for tumor control. Some of the innate mechanisms that have evolved against infections with microorganisms and viruses are proving to be active against cancer cells but require better understanding of how they are activated and what inhibitory mechanisms may need to be targeted. This is particularly so for inflammasomes which have evolved against many different organisms and which recruit a number of cytotoxic mechanisms that remain poorly understood. Equally important is understanding of where these mechanisms will fit into existing treatment strategies and whether existing strategies already involve the innate killing mechanisms.

## 1 INTRODUCTION

The introduction of drugs that targeted the *BRAFV600E* mutation in the MAPK signal pathway and advances in immunotherapy based on blockade of immune checkpoints on lymphocytes ushered in a new era in treatment of melanoma (Luke, Flaherty, Ribas, & Long, 2017). Despite these advances, not all patients respond to such treatments and even in responding patients therapy resistance occurs in the majority of patients. Multiple causes of resistance to immunotherapy have been defined (Ribas & Wolchok, 2018; Sharma, Hu‐Lieskovan, Wargo, & Ribas, 2017) including defects in antigen presentation (Sade‐Feldman et al., 2017) and low T‐cell infiltration into tumors (Li et al., 2019). Similarly, resistance to targeted therapies involves a diverse array of causes including reactivation of the MAPK pathway (Song, Piva, et al., 2017) and epigenetic changes (Hugo et al., 2015; Shaffer et al., 2017).

Recent studies on resistance mechanisms have placed increasing importance on the plasticity of some melanoma and adaptive changes induced by treatment (Bai, Fisher, & Flaherty, 2019). These concepts have led to classifications based on different states of differentiation (Tsoi et al., 2018) and that treatment resistance can involve dedifferentiation of melanoma to relatively undifferentiated states (Tsoi et al., 2018). These effects of therapy with MAPKi had been shown previously in melanoma (Hugo et al., 2015). Similarly, resistance to immunotherapy had been shown in previous studies to be associated with dedifferentiation and loss of melanoma antigens due to TNF production by T cells responding to the tumor (Landsberg et al., 2012). Similar findings were reported in adoptive T‐cell therapy in melanoma with loss of differentiation antigens MART1 and gp100 (Mehta et al., 2018).

An important finding in studies on melanoma resistance was that certain dedifferentiated resistant melanoma was nevertheless sensitive to anti‐cancer drugs when their expression profiles were matched to drugs in a pharmacogenomics portal (Seashore‐Ludlow et al., 2015). In particular, dedifferentiated melanoma was sensitive to ferroptosis‐inducing drugs. These studies have raised questions as to whether other innate cytotoxic mechanisms may have different resistance mechanisms that can be targeted particularly in melanoma not responding to targeted or immunotherapy. The following sections review the studies on ferroptosis and then review evidence that pyroptotic cell death induced by inflammasomes may also provide novel approaches against resistant melanoma.

## 2 FERROPTOSIS AS A MODEL OF INNATE CELL DEATH MECHANISMS IN CANCERS

Ferroptosis is a non‐apoptotic form of cell death resulting from iron‐dependent lipoxygenase enzyme peroxidation of polyunsaturated fatty acids in cell membranes (Dixon, 2017). These enzymes are normally inhibited by glutathione‐dependent anti‐oxidants such as glutathione peroxidase 4 (GPX4; Yang et al., 2016). Ferroptosis can therefore be induced by inhibition of GPX4. The production of GPX4 is dependent on membrane transporters that transport cysteine needed for production of glutathione. The transporters can be inhibited by drugs like erastin or sorafenib and GPX4 itself by RSL3 and FIN56. Anti‐oxidants like ferrostatin‐1 also inhibit ferroptosis. The significance of GPX4 in treatment resistance of cancer cells was revealed in studies on drug‐tolerant persister cells from several types of cancer that were found to be vulnerable to inhibition of GPX4 (Hangauer et al., 2017; Viswanathan et al., 2017). Further interest in ferroptosis was generated by the discovery that immunotherapy with anti‐CTLA4 and anti‐PD1 was inhibited by the anti‐oxidant liprostatin‐1 (Wang et al., 2019), indicating that although ferroptosis was an endogenous cytotoxic mechanism, it could also be induced by T cells. The mechanism of induction of ferroptosis by the T cells appeared to involve interferon (IFN)‐γ‐mediated downregulation of the glutamate–cystine transporter system required for production of GPX4 (Zitvogel & Kroemer, 2019).

## 3 INFLAMMASOME‐INDUCED CELL DEATH IN CANCERS

These studies on ferroptosis‐induced death in cancer cells that are resistant to treatment raise the possibility that other innate cell death mechanisms may also be recruited against melanoma. These include regulated pathways such as necroptosis and pyroptosis. Necroptosis is a programmed form of necrosis showing morphological features similar to necrosis that is dependent on activation of the receptor‐interacting serine/threonine kinases (RIPK)1, RIPK3 and mixed lineage kinase domain‐like pseudo‐kinase (MLKL). The latter is recruited to phosphotidylinosites and oligomerizes in the plasma membrane (Kaczmarek, Vandenabeele, & Krysko, 2013; Tang, Kang, Berghe, Vandenabeele, & Kroemer, 2019). It does not involve caspases but can be induced by death receptor ligands such as TNF and Fas when caspase‐8 is inhibited or at low expression levels. It was found that RIPK3 mRNA and protein were absent or poorly expressed in most metastatic melanoma (Geserick et al., 2015) which is a limitation in targeting necroptosis in new treatment initiatives.

Pyroptosis is also a regulated form of cell death resulting from activation of inflammasomes as outlined in several reviews (Lee & Kang, 2019; Moossavi, Parsamanesh, Bahrami, Atkin, & Sahebkar, 2018; Xia et al., 2019). Inflammasomes have been mainly of interest in defense of cells against infections. However, their association with inflammation and cell death has created interest in their involvement in a wide range of diseases such as obesity, dementia, diabetes, and cancers (Guo, Callaway, & Ting, 2015).

There are many different types of inflammasomes but in general they are cytosolic protein complexes composed of sensors that recognize microbial components and products of cell injury, an adaptor protein apoptosis‐associated speck‐like protein containing a caspase recruitment domain (ASC), and caspase‐1 that binds to ASC. The formation of such a complex leads to the activation of caspase‐1, which induces the cleavage and secretion of pro‐inflammatory cytokine interleukin‐1β (IL‐1β) and IL‐18 (Lamkanfi & Dixit, 2014). The sensors are categorized according to their structural characteristics into nucleotide‐binding domain‐like receptors (NLRs), and absent in melanoma 2 (AIM2)‐like receptors (ALRs). The NLR sensors can be specific for certain bacterial products such as the lethal toxin of *Bacillus anthracis* in NLRP1 and bacterial flagellin in NLRC4. NLRP3 appears less specific and can be activated by crystalline structures such as uric acid crystals and a range of bacterial products and viruses (Lamkanfi & Dixit, 2014).

Activation of NLRP3 is believed to occur in two stages with an initial priming step resulting from activation of NF‐kB, for example, by toll‐like receptors (TLR) which increases the levels of proteins in the complex. Deubiquitination of NLRP3 by deubiquitinating enzymes (Py, Kim, Vakifahmetoglu‐Norberg, & Yuan, 2013) and phosphorylation steps mediated by c‐Jun terminal kinase (JNK1; Song, Liu, et al., 2017) are also involved. Following the priming step, activation of the complex can occur in response to a number of stimuli including particulate matter, bacterial and viral products, and ATP. It was proposed that the common mechanism may be potassium (K^+^) efflux and low K levels. This would be consistent with the proposed mechanism of activation by the antibiotic nigericin, which is believed to cause efflux of K^+^ in exchange for H^+^ (Próchnicki, Mangan, & Latz, 2016). Another protein involved in assembly of the inflammasome is the enzyme NEK7 that binds to the so‐called LRR domain of NLRP3. This forms a connection with adjacent NLRP3 proteins allowing oligomerization to occur (Nozaki & Miao, 2019).

The AIM2 sensor was discovered in experiments designed to suppress tumorigenicity of melanoma cells by transfer of chromosome 6 from normal cells. One of the resulting differentially expressed genes was AIM2 which belonged to the family of interferon‐inducible genes (DeYoung et al., 1997). Subsequent studies have shown that it binds to double‐stranded DNA (dsDNA) in the cytosol either released from the nucleus or from bacteria and DNA viruses (Lugrin & Martinon, 2018). Once bound to DNA, it forms a helical structure with ASCs by their pyrin domain (PYD) and caspase‐1 then binds to ASC proteins via the CARD domains (Wang & Yin, 2017). In addition to recognition of dsDNA, AIM2 can also recognize endogenous retroviruses (Sharma, Karki, & Kanneganti, 2019)that leads to activation of endogenous IFN pathways in immune responses (Chuong, Elde, & Feschotte, 2016). AIM2 is subject to degradation by autophagy once it is bound by tripartite motif protein 11 (TRIM11). This is viewed as an autoregulatory mechanism to control inflammation (Liu et al., 2016). Immunohistochemistry studies have shown high levels of AIM2 in inflammatory skin disorders (de Koning et al., 2012) and in primary melanoma (de Koning, van Vlijmen‐Willems, Zeeuwen, Blokx, & Schalkwijk, 2014).

## 4 CYTOKINES AND CELL DEATH RESULTING FROM ACTIVATION OF THE INFLAMMASOME

The key downstream events of activation of inflammasomes are activation of caspase‐1 which converts pro‐interleukin (IL)‐1β and pro‐IL‐18 into the active cytokines IL‐1β and IL‐18. Caspase‐1 also cleaves a protein called gasdermin D. Gasdermin D is a member of a family of conserved proteins that includes gasdermin A, B, C, D, E, and DFNB59 (Orning, Lien, & Fitzgerald, 2019). They have an N‐terminal pore forming domain (PFD) composed of 242 amino acids (aa) connected by a 43‐aa linker to a 199‐aa carboxy‐terminal domain. After cleavage by caspase‐1, the N‐terminal PFDs oligomerize and integrate into the cell membranes to form large diameter pores of 10‐15nm which allows entry of solutes and disruption of the cell as well as release of IL‐1β and IL‐18. Most of the gasdermins have pore‐forming ability that is held in check by their C‐terminal domain (Kovacs & Miao, 2017). Gasdermin E can be cleaved by caspase‐3 and can thereby increase apoptosis induced by intrinsic pathways. Both gasdermin D and E also permeabilize mitochondrial membranes and provide a link between these two death pathways (Rogers et al., 2019). The central role of gasdermins in pyroptosis has led to the proposal that it be referred to as gasdermin mediated programmed necrotic cell death (Shi, Gao, & Shao, 2017).

## 5 GOOD AND BAD ASPECTS OF INFLAMMASOME ACTIVATION IN CANCERS

The activation of inflammasomes while potentially inducing cell death in cancers may also have tumor‐promoting properties due to induction of chronic inflammation. The potential effects on individual tumors have been reviewed elsewhere (Liu et al., 2015; Moossavi et al., 2018; Xia et al., 2019). Inflammasomes were found to be inactive in primary melanoma but constitutively active in high‐grade metastatic melanoma (Dunn, Ellis, & Fujita, 2012; Okamoto et al., 2010). Nodular melanoma was reported to be more common in certain polymorphisms of NLRP3 and NLRP1 (Verma et al., 2012). One of the main products of inflammasome activation is IL‐1β which has been implicated in promotion of lung cancers. This was based on a significant reduction of lung cancers in the CANTOS trial on 10,061 patients with cardiovascular disorders and high C‐reactive proteins (CRPs) who were randomized to placebo or different doses of canakinumab, an antibody against IL‐1β. The trial was positive in terms of reduction of cardiovascular events. In addition, a retrospective analysis showed a highly significant reduction in incidence of lung cancers in patients receiving the two higher doses of canakinumab (Ridker et al., 2017).

Retrospective analysis of an immunotherapy trial in melanoma also raised questions about association of high CRPs with suppression of responses against immunotherapy with anti‐CTLA4. This trial compared results of immunotherapy with tremelimumab (anti‐CTLA4) with those against standard chemotherapy. The intent‐to‐treat results were negative (Ribas et al., 2013) but a retrospective analysis showed that when patients with high CRPs were excluded, the patients treated with tremelimumab did have better survival than the chemotherapy treated patients (Marshall, Ribas, & Huang, 2010). A small phase 2 study on 37 patients treated with interferon and tremelimumab showed increased survival was associated with low baseline CRP levels (Tarhini et al., 2012). CRPs are induced in the liver by IL‐6 which is upregulated by activation of NF‐kB by IL‐1β so this is again indirect evidence for an adverse effect of activated inflammasomes on immune responses against melanoma.

In contrast, the importance of inflammasomes in immune responses against cancers received strong support from studies on an innate immune checkpoint referred to as transmembrane protein 176B (TMEM176B). TMEM176B regulates Ca^2+^‐dependent K^+^ channels and thereby prevents development of low levels of K^+^ in the cytosol that is a strong activator of NLRP3 inflammasomes. Block of this checkpoint in models of several murine tumors allowed activation of NLRP3 inflammasomes and enhanced immune responses against the tumors. Immunotherapy with anti‐PD1 and anti‐CTLA4 against EG7 lymphomas was also enhanced by blockade of TMEM176B. Importantly, they found that human melanoma tumors responding to anti‐CTLA4 and anti‐PD1 had upregulation of 15/16 inflammasome‐related genes that was not detected in melanoma that did not respond to ICB (Segovia et al., 2019). These gene expression differences were not observed in pretreatment samples. Pharmacological inhibition of TMEM176B was associated with increased infiltration of tumors by CD8 T cells. Similar enhanced anti‐PD1 responses were seen against mice bearing a murine melanoma. The authors suggested that TMEM176B may be a useful marker to predict responses to ICB immunotherapy with high levels being associated with poor responses (Segovia et al., 2019).

Dendritic cells (DCs) provide the essential link between innate and adaptive immunity and evidence suggests the CD141+ (cDC1) subset are of critical importance in generation of CD8 T‐cell responses (van der Aa, van Montfoort, & Woltman, 2015; Roberts et al., 2016). Inflammasomes are known to be expressed in DCs but whether they are critical for directing type 1 responses is not known (Ferreira et al., 2017). AIM2 in plasmacytoid DCs in lung carcinoma was considered responsible for immunosuppression associated with IL‐1 alpha production (Sorrentino et al., 2015). NLRC4 inflammasome in cDC1 was the target for dabrafenib activation (Hajek et al., 2018) but its role in treatment responses remains to be studied. The need for priming steps for activation of inflammasomes may provide a strategy for selective activation of cells in the immune system and thereby increase immune responses against cancers. Recent studies have shown impressive responses in patients that had failed anti‐PD1 treatment when treated with TLR9 agonists. TLR9 expression is confined to plasmacytoid DCs and CD141 DCs so that activation of inflammasomes by these agonists would be selective for the immune cells (Poh, 2018; Ribas et al., 2018).

## 6 DEVELOPING A UNIFYING HYPOTHESIS

**Figure 1:**
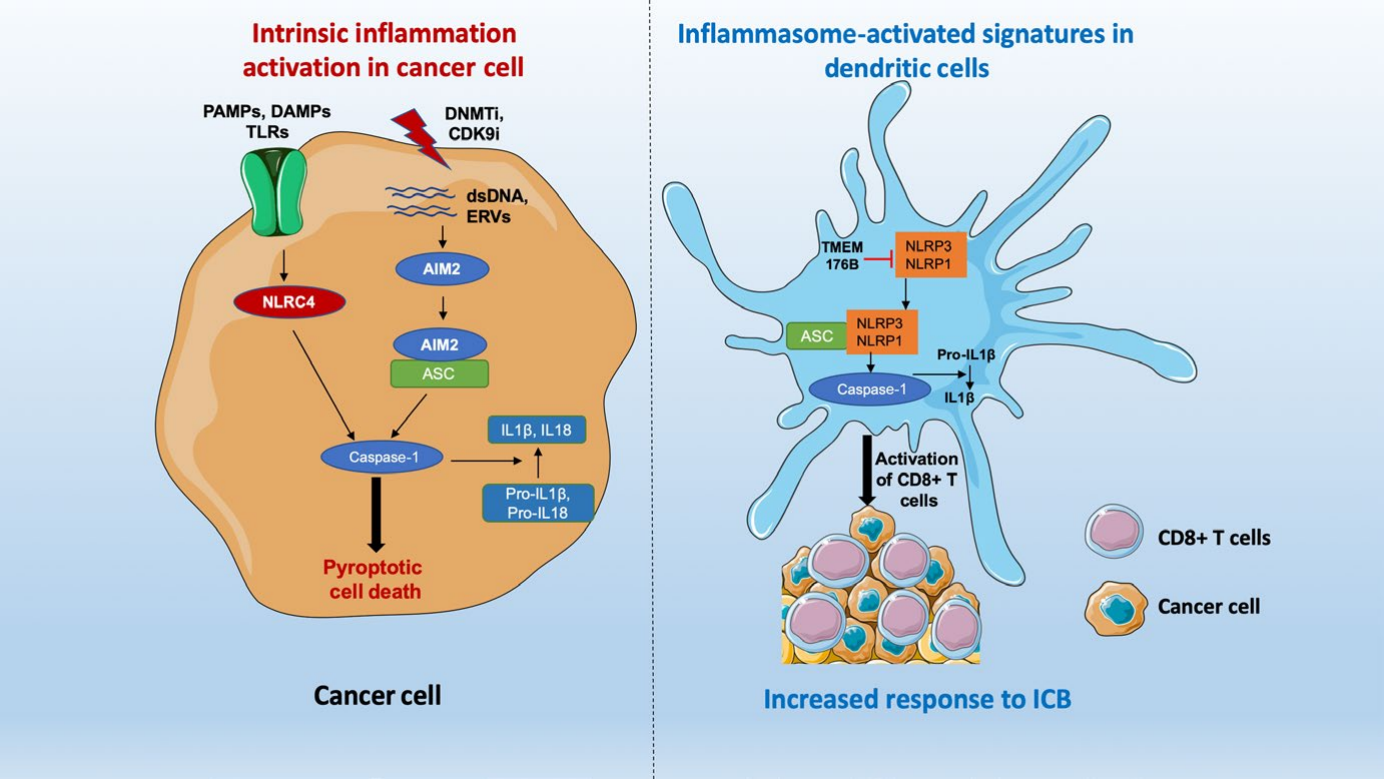
Possible mechanisms of pyroptotic cell death through inflammasome activation. Epigenetic drugs such as DNA methyltransferase inhibitor (DNMTi) and CDK9 inhibitors (CDK9i) can activate ERVs and dsDNA in the cancer cells. Inflammatory protein AIM2 binds dsDNA and subsequently activates caspase‐1 through forming a complex with ASC protein. Whereas, DAMPs, PAMPs binds to TLRs on the plasma membrane and subsequently activate inflammatory sensor NLRC4, which can directly activate caspase‐1. Activated caspase‐1 directs pyroptotic cell death and converts IL‐1β, IL18 from inactive Pro‐IL‐1β, Pro‐IL18 that releases from the cells to drive inflammation (left panel). Intrinsic inflammatory gene signatures in the dendritic cells or macrophages augment response to ICB. TMEM176B is a negative regulator of the inflammatory proteins such as NLRP3 and NLRP1. Blocking TMEM176B by small molecule inhibitor leads to activation of caspase‐1 and IL‐1β. This promotes the recruitment of CD8+ T cells in the tumor microenvironment thus enhancing the therapeutic response of ICB (right panel) (modified from Segovia et al., 2019). DAMPs, damage‐associated molecular patterns; ERVs, endogenous retroviruses; ICB, immune checkpoint blockade; PAMPs, pathogen‐associated molecular patterns; TLR, toll‐like receptor.

These and previous studies indicate that inflammasomes have diverse roles in cancer with some cancers benefiting from IL‐1β and IL‐18, whereas in others, the IL‐1 signaling pathway promoted cancer growth (Karki & Kanneganti, 2019; Karki, Man, & Kanneganti, 2017). We suggest the best unifying hypothesis is that the outcomes depend on whether inflammasome activation is predominantly within the tumor or the immune cells. In the Segovia et al. study, the benefits appeared to be clearly focused on immune responses to the tumors and the TMEM176B checkpoint did not work through changes in the tumor cells. It also appeared that the effects of blocking this checkpoint were mainly evident in immunogenic tumors and not in tumors unresponsive to immunotherapy (Segovia et al., 2019; Figure 1).

## 7 CLUES FROM THE CANCER GENOME ATLAS (TCGA) MELANOMA DATA

**Figure 2:**
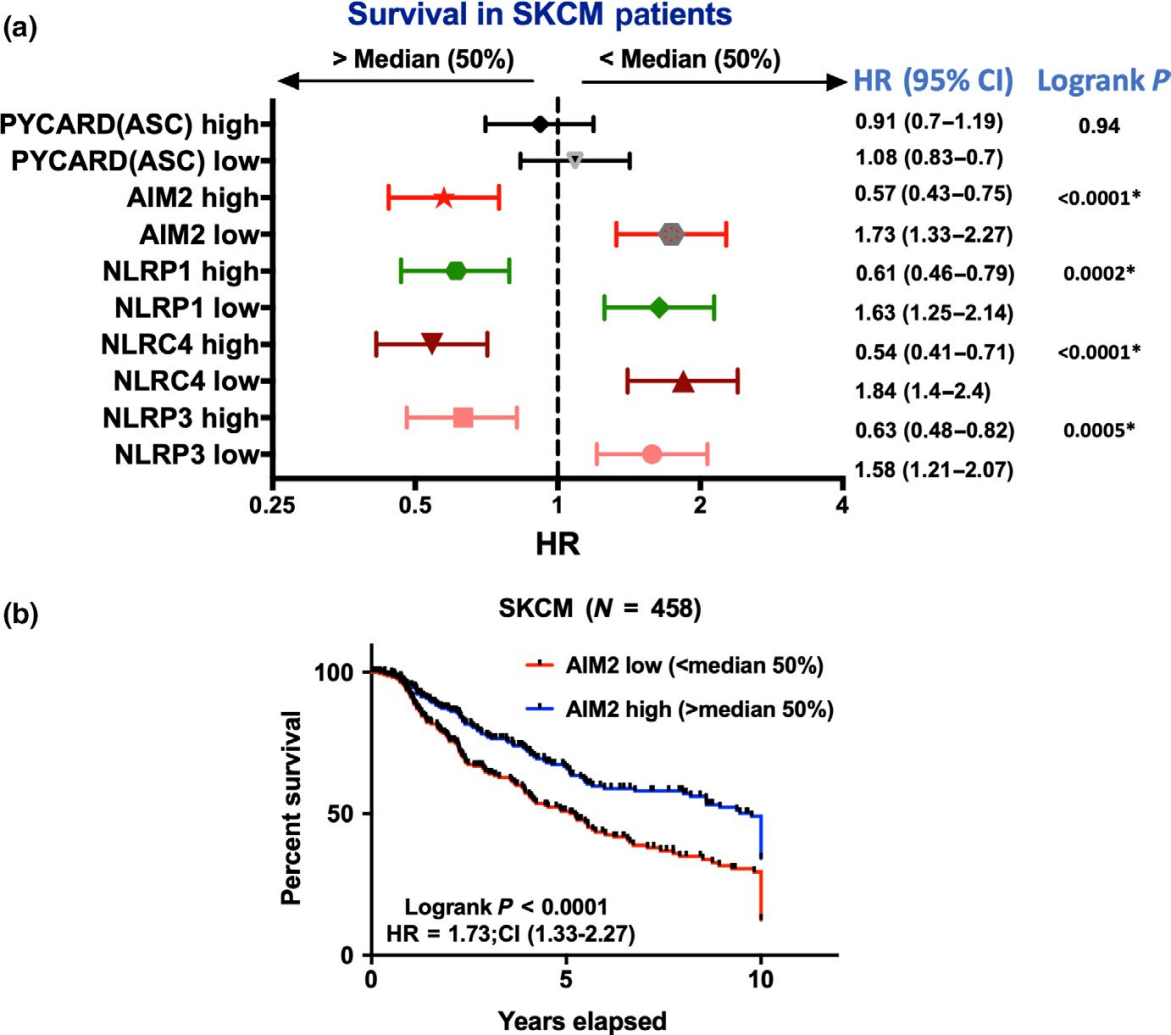
Low expression of inflammasome mediators is associated with poor prognosis in melanoma. RNA‐seq data of skin cutaneous melanoma (SKCM) patients were retrieved from TCGA database (*N* = 458). Patients were dichotomized based on median expression (>median = high; <median = low) of the corresponding genes. (a) A forest plot was generated based on the computed hazard ratio (HR) and 95% confidence intervals (CI) of survival for each gene. Logrank *p* value refers to the significance of the overall survival adjusted to 10 years. (b) A KM‐plot is showing overall survival of SKCM patients based on AIM2 median expression

Studies on information in the TCGA have proven useful in identifying subgroups of patients with different outcomes. It was reasoned that high levels of proteins associated with inflammasomes may identify their effect on survival. RNA‐seq data from 458 patients with cutaneous melanoma were interrogated for associations of inflammasome proteins with patient survival by comparing outcomes in patients with high (>median) versus low (<median) levels. The forest plots shown in Figure 2 indicate that the RNA‐seq expression levels, above or below the median, of AIM2, NLRP3, NLRP1, and NLRC4 show a strong positive correlation with survival. Expression of ASC (PYCARD) adaptor proteins was not related to survival.

**Figure 3:**
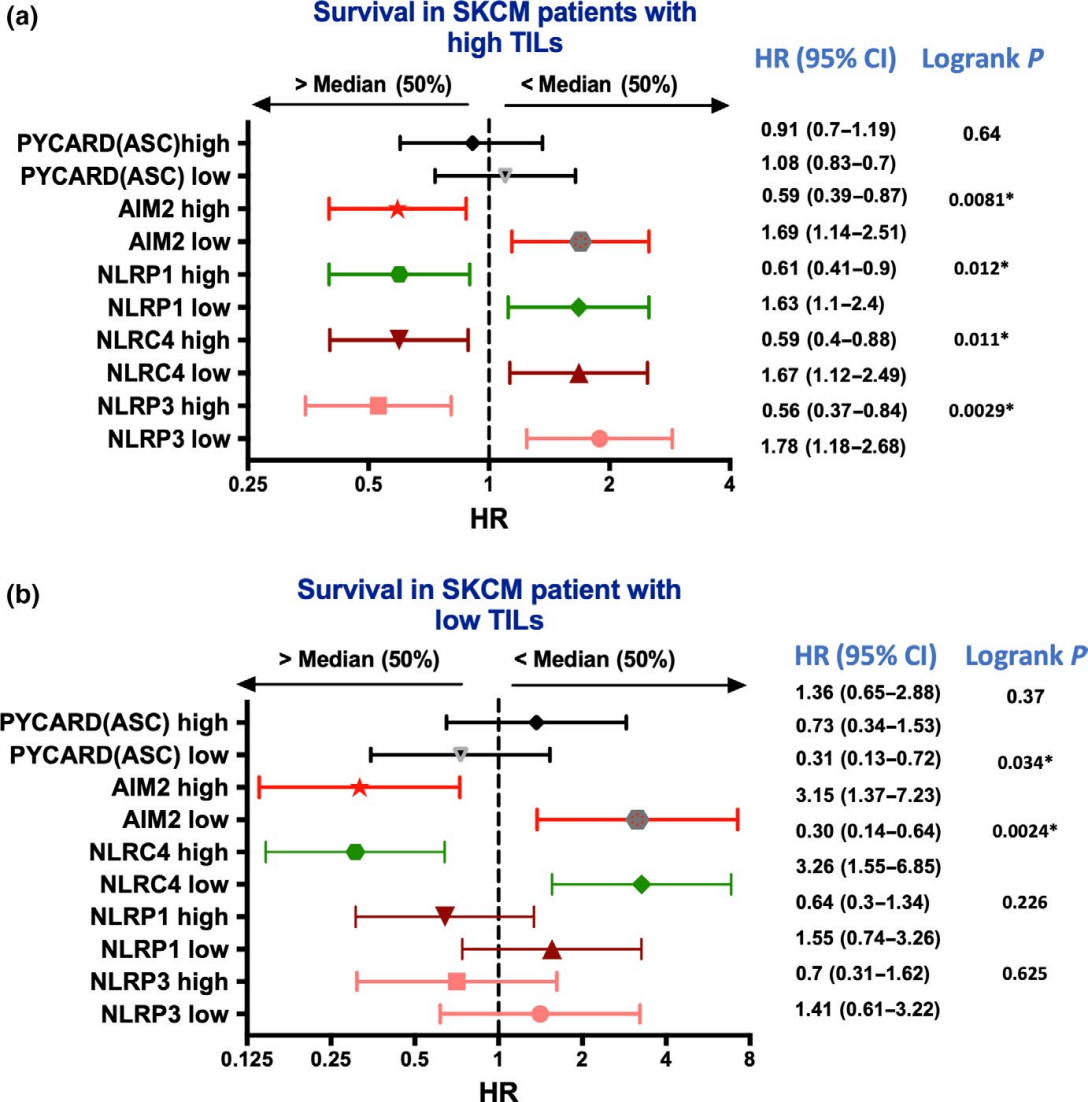
Association of TILs level and gene expression of the inflammasome mediators with overall survival in melanoma. (a) Skin cutaneous melanoma (SKCM) patients were separated based on the median expression of the corresponding gene and the TILs level (>5% refers to high TILs). TILs proportion in the tumor were retried based on deep learning pathology images (Saltz et al., 2018). (b) Similarly, SKCM patients were stratified based on low TILs level (<5% refers to low TILs) and median expression of the selected gene. Forest plot refers to the HR with 95% CI and logrank *p* value were calculated for overall survival. Statistical analysis was performed in GraphPad prism, and *p* < .05 refers to significance of overall survival

As discussed above, the outcome of inflammasome activation may depend on whether inflammasomes are activated in immune cells or in cancer cells. TCGA analyses shown in Figure 2 did not discriminate between these effects but we reasoned that if the results were due to effects related to activation in immune cells this would be most evident in melanoma with high levels of T cells infiltrating lymphocytes (TILs). The effects on survival were therefore compared in patients with <5% TILs and those with >5% TILs using information reported elsewhere (Chen, Khodadoust, Liu, Newman, & Alizadeh, 2018; Saltz et al., 2018). The improvement in survival with high levels of inflammasome receptors NLRP1 and NLRP3 was abrogated in patients with low TILs (Figure 3a). In contrast, the improved survivals seen with high AIM2 and high NLRC4 were retained in patients with high or low TILs score suggesting the beneficial effect might be intrinsic to melanoma cells and independent of TILs level (Figure 3b).

NLRP1 activation is of particular interest in that recent studies suggest that it is held in an inactive state by the serine dipeptidyl peptidases (DPP)8 and 9 and that inhibitors of these dipeptidases result in activation of NLRP1.(Zhong et al., 2018) Preclinical studies with non‐specific inhibitors of DPP8/9 (also known as PT100 or talabostat) had previously shown that these inhibitors had anti‐tumor effects that appeared mainly related to effects on the immune system(Adams et al., 2004).

NLRC4 is believed to be activated by the NOD‐like apoptosis‐inducing protein (NAIP) sensor which binds a number of different gram‐negative bacteria. It can bind directly to caspase‐1 rather than through binding to ASC and can activate caspase‐8. It has also been associated with high levels of IL‐18 (Duncan & Canna, 2018). In a murine model of melanoma, NLRC4 was found to suppress tumor growth by non‐inflammasome activation involving interferon‐gamma production from tumor‐associated macrophages. The latter were prominent in primary melanoma but not in metastatic melanoma (Janowski et al., 2016). AIM2 is activated by dsDNA from pathogens or damaged nuclei. It was also activated by IFN in a network involving endogenous retroviruses upstream of AIM2 (Chuong et al., 2016).

### 7.1 C‐reactive proteins as markers of harmful inflammasome activation

IL‐1β is a very pleiotropic cytokine that has effects that can promote or inhibit growth of tumors (Bent, Moll, Grabbe, & Bros, 2018). It can also have downstream effects resulting in activation of NF‐kB and production of cytokines like IL‐6 that activate acute phase proteins like CRP (Slaats, Ten Oever, van de Veerdonk, & Netea, 2016). CRP levels are recognized markers of inflammation, and several studies have identified high CRP levels (>10 mg) to be associated with an adverse prognosis in melanoma. A comprehensive study on 1,144 patients found that approximately 10% of patients had elevated levels, and this was an adverse prognostic indicator at all stages of the disease (Fang et al., 2015). Sequential studies also showed that increased levels could predict recurrences.

**Figure 4:**
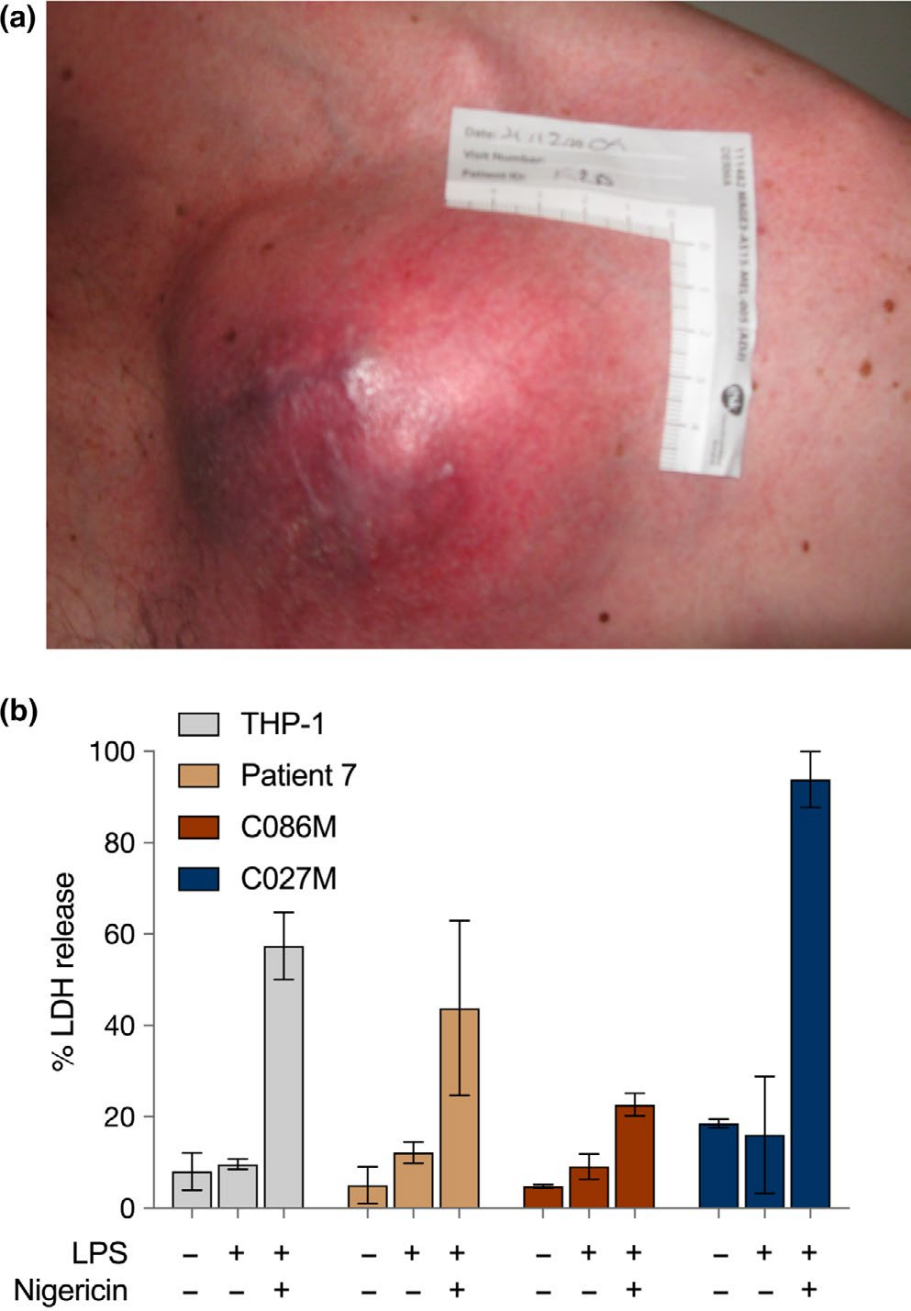
High expression of inflammatory gene signatures induced cell death in patient‐derived melanoma cells. (a) Clinical appearance of melanoma in patient 7. (b) Melanoma with strong inflammasome RNA‐seq signatures (patient 7, C027M) display nigericin‐dependent lytic cell death. The THP‐1 human monocytic cell line was used as a positive control; C086M human melanoma cell line has weaker inflammasome RNA‐seq signatures

Certain forms of metastatic melanoma when they involve subcutaneous sites can be associated with clinical appearances of marked inflammation and systemic symptoms of fevers, anorexia, and weight loss. The clinical appearance of one such metastasis is shown in Figure 4a. Melanoma cultures from this patient (patient 7) were shown to produce high levels of a range of cytokines including IL‐1β, IL‐6, IL‐8, and VEGF, which were associated with constitutive activation of NF‐kB (Gallagher et al., 2014). The BET protein inhibitor, I‐BET151, was very effective in inhibiting the cytokine production in vitro and this or more recently developed BET protein inhibitors (Xu & Vakoc, 2017) may have a role against the severe forms of inflammatory melanoma (Gallagher et al., 2014). We have subsequently shown that such melanoma can be associated with marked global hypomethylation of DNA and expression of inhibitory ligands such as PD‐L1 (Chatterjee et al., 2018; Emran et al., 2019). These studies are consistent with an adverse effect of inflammasome activation in melanoma due to both growth‐promoting effects and immunosuppression from low‐grade chronic inflammation.

## 8 DIFFERENTIATION STATE OF MELANOMA AS DETERMINANTS OF INFLAMMASOME ACTIVATION

Part of the paradigm introduced by the studies of Tsoi et al. was that different states of differentiation of melanoma have different sensitivity to particular treatments. In the case of ferroptosis, the susceptible melanoma cells appeared to have a neural crest‐like phenotype. Triggers for cell death were agents that reduced or inhibited the anti‐oxidants like GPX4 that inhibit the lipoxygenase enzymes (Tsoi et al., 2018). In the case of NLRP3 in normal cells, a priming step increases the concentration of proteins in the complex and also involves deubiquitination and phosphorylation steps referred to above. Whether a priming step is needed in cancer cells is not clear as the malignant state may provide this priming step. This was supported by studies on melanoma that showed constitutive activation of NALP (NLRP3) inflammasomes in melanoma occurred in late‐stage metastatic melanoma but not in intermediate or early‐stage melanoma. The inflammasomes in intermediate stage melanoma could however be activated by exposure of the melanoma to IL‐1 (Okamoto et al., 2010). Studies on melanoma cell lines suggest that sensitivity to the nigericin activator of NLRP3 was associated with high levels of NLRP3 complex proteins and other inflammasome sensors (unreported data) but whether levels are associated with different states of differentiation remains to be studied (Figure 4b).

The need for priming steps for activation of inflammasomes may provide a strategy for selective activation of cells in the immune system and thereby increase immune responses against cancers. Recent studies have shown impressive responses in melanoma patients that had failed anti‐PD1 treatment when treated with TLR9 agonists. TLR9 expression is confined to plasmacytoid DCs so that activation of inflammasomes by these agonists would be selective for the immune cells (Poh, 2018; Ribas et al., 2018).

## 9 REPURPOSING DRUGS TO ACTIVATE INFLAMMASOMES

Recent studies showing that the serine dipeptidases DPP8/9 inhibit activation of NLRP1 has put renewed interest in past studies on inhibitors of these peptidases (like talabostat) as anti‐tumor agents (Eager et al., 2009). In particular, studies on AML have shown that with appropriate selection a large proportion of AML lines can be killed by these and more specific inhibitors of DDP8/9 (Johnson et al., 2018). In addition, a number of chemotherapy agents that have modest activity against cancers such as melanoma may have ancillary effects on inflammasomes. For example, paclitaxel was shown to activate TLR in macrophages and to prime macrophages for NLRP3 inflammasome activation by ATP or nigericin (Son, Shim, Hwang, Park, & Yu, 2019). IL‐1β production was totally dependent on presence of NLRP3. Doxorubicin was found to induce pyroptosis in several melanoma lines in vitro particularly when autophagy was inhibited by chloroquine (Yu et al., 2019).

Autophagy is a critical regulator of inflammasome activation by removal of endogenous signals that would otherwise activate inflammasomes. It is also critical for degradation of inflammasome components and may be a physiological feedback mechanism to control inflammation (Harris et al., 2017; Seveau et al., 2018). Chloroquine and several derivatives are currently in clinical trials with chemotherapy but whether inflammasomes were involved is not known (Amaravadi, Kimmelman, & Debnath, 2019; Rebecca et al., 2019). Temozolomide was shown to induce responses in 3 patients who had failed immunotherapy with pembrolizumab (Swami et al., 2019) but whether activation of inflammasomes was involved is an intriguing possibility. A particularly interesting report was the activation of inflammasomes by the BRAFV600E targeting drug dabrafenib (Hajek et al., 2018). As reviewed elsewhere (Hersey, Tiffen, & Gallagher, 2019), the cause of fevers induced by dabrafenib has long been a puzzle and this report provides a plausible explanation as well as opening up new areas of research such as whether off‐target effects of dabrafenib on inflammasomes may increase immune responses against melanoma.

Drugs that demethylate DNA in the nucleus like decitabine or azathioprine may activate AIM2 inflammasomes. Endogenous retroviral elements (ERV) in particular were shown to activate AIM2, and further studies are needed to examine whether cell death induced by these drugs is associated with activation of AIM2 (Chuong et al., 2016). Inhibitors of CDK4/6 were also reported to expose ERV elements and potentially be involved in the activation of immune responses by these drugs (Goel et al., 2017). CDK9 inhibitors are proving to be another class of epigenetic regulators that can reactivate genes silenced in heterochromatin to an active state in euchromatin by phosphorylation of BRG1 in the SWI/SNF complex (Zhang et al., 2018). These brief references indicate there is much scope for examining the role of inflammasomes in the activity of these agents (Figure 1).

## 10 CONCLUSION

Pyroptosis induced by activation of inflammasomes is potentially an additional cell death mechanism that is not subject to the obstacles that limit apoptosis and other forms of cell death. Nevertheless, the role of inflammasomes in cancer and melanoma in particular is complicated in that the inflammatory response generated may promote tumorigenesis as suggested for lung carcinoma in the CANTOS trial. There is also indirect evidence that inflammation from activation of inflammasomes may be immunosuppressive and inhibit immunotherapy induced by ICB. In contrast, data from TCGA analyses can be interpreted to suggest that inflammasomes in melanoma may be associated with improved prognosis.

We suggest these different interpretations may depend on whether there is chronic activation of inflammasomes in the tumor itself giving rise to tumor promotion and immunosuppression or whether the site of inflammasome activation is in the immune system (particularly DCs) as shown in Figure 1. The latter may amplify the inherent cytotoxic mechanisms and lead to tumor control. The TCGA data, though indirect, would be consistent with this interpretation and is supported by studies in murine models.

Should these interpretations prove valid a two‐pronged treatment approach might be considered that would involve selection of patients with evidence of chronic activation of inflammasomes in their tumor such as high CRP levels and treatment with agents that limit inflammation such as inhibition of circulating IL‐1β and inhibition of NF‐kB by use of BET protein inhibitors. On the other hand, should favorable outcomes depend on activation of inflammasomes in the cells of the immune system, selection of patients might be based on those not responding to ICB and treatment with agents that activate the inflammasome. There is very limited understanding of the role of inflammasomes in relation to different subsets of DCs and whether activation of different inflammasomes has different treatment outcomes. New initiatives that target inflammasomes in the immune system might include blockade of the TMEM176B checkpoint that limits activation of inflammasomes in T cells as reviewed above. There may also be scope for treatments with drugs that have selectivity for particular inflammasomes. The targeting of NLRP1 inflammasomes by DPP8/9 inhibitors or the targeting of NLRC4 inflammasomes by dabrafenib may be examples of such agents as well as the well‐known TLR9 agonists mentioned above. Blocking autophagy to increase activation of inflammasomes might also be worthy of investigation in drugs shown in past studies to have only modest benefits against melanoma.

## CONFLICT OF INTEREST

The authors have no conflict of interest in relation to this work.
